# Shifting Focus to Quality: An Innovative Modeling Approach Includes Processing History for Rubber Part Quality Simulation

**DOI:** 10.3390/polym17020149

**Published:** 2025-01-09

**Authors:** Roman Christopher Kerschbaumer, Georg Weinhold, Florian Leins, Martin Traintinger, Michaela Hornbachner, Maurício Azevedo, Thomas Lucyshyn

**Affiliations:** 1 Polymer Competence Center Leoben GmbH, Sauraugasse 1, 8700 Leoben, Austria; roman.kerschbaumer@pccl.at (R.C.K.);; 2 MAGMA Giessereitechnologie GmbH, Kackertstrasse 16–18, 52072 Aachen, Germany; 3 Department of Polymer Engineering and Science, Polymer Processing, Montanuniversitaet Leoben, Franz-Josef Strasse 18, 8700 Leoben, Austria; thomas.lucyshyn@unileoben.ac.at

**Keywords:** rubber, normalized degree of cure, average curing speed (ACS) model, simulated part quality, innovative modeling approach, processing history

## Abstract

An innovative modeling approach for the simulative description of the part quality of rubber materials, including the processing history, is presented in this paper. This modeling approach, the so-called average curing speed (ACS) model, is based on the degree of cure and the average curing speed instead of the conventionally considered temperature approach. Such approach neglects the processing history by calculating only the degree of cure. Thus, the correlation with part quality has to be performed either after the simulation or with the aid of other numerical analysis programs. Instead, by applying the ACS model, the key advantage is that the processing history is already taken into account during the filling and curing simulation, demanding a single calibration step with quality information to be able to calculate the part quality. For this purpose, parts were manufactured at mold temperatures ranging from 140 °C to 170 °C and degrees of cure from 24% to 99% via compression molding and subsequently the permanent deformation, i.e., the compression set (CS), of each part was analyzed. The CS results show that one and the same degree of cure; for example, 80%, which was defined on the basis of reaction isotherms, causes an almost twofold higher CS value for parts manufactured at 170 °C. Consequently, considerable deviations may occur when real part qualities are correlated with degrees of cure from simulations with common state-of-the-art kinetic models. By applying the ACS model, it was demonstrated that this challenge could be solved. Parts manufactured by compression molding exhibited the same quality as those simulated with the ACS model. Finally, this innovative modeling approach (fully implemented in the SIGMASOFT^®^ v6.0 simulation routine) provides enormous potential for understanding local differences in the quality of rubber parts, being an ideal tool for optimizing rubber parts through simulation routines.

## 1. Introduction

High-performance elastomer products are generally preferred for applications subjected to extreme dynamic or cyclical loads, such as seals, damping elements, hoses, and tyres. In order to meet the high demands placed on the material in this respect, rubber compounds consist of a large number of ingredients [1]. The essential ones include: (i) the base polymer, (ii) active fillers such as carbon black or silica, and (iii) the curing system, generally based on sulfur or peroxide. Sulfur vulcanization is the oldest and remains the most widely considered system for curing of unsaturated elastomers [2]. In this sense, an effective and rapid curing reaction of rubber materials with sulfur occurs only in the presence of accelerators and activators. Both influence the kinetic parameters (especially the curing speed), the processing safety of the rubber compound, and the amount of sulfur needed for the optimal crosslinked structure of the elastomeric product. Thus, the type and amount of accelerator and the ratio of the accelerator to sulfur in the rubber compound impacts the final part properties, because of its influence on the sulfur chain composition [2,3,4,5,6,7,8]. In this context, a distinction is made between poly-, di-, and monosulfidic crosslinks, which are formed during the vulcanization process. In addition to the selected crosslinking system, the process parameters also have a significant influence on the type of crosslinks formed in the product. For example, elevated mold temperatures and/or prolonged vulcanization times result in a dissociation reaction of the polysulfidic crosslinks. These are converted to di- and monosulfidic bonds, resulting in a distinct crosslink length distribution, an increased crosslink density, and finally leading to a different part quality [6,9,10,11,12,13,14,15,16,17,18,19]. Overall, it can be concluded that (i) the sulfur crosslinking only proceeds efficiently if accelerators and activators are used, as this optimizes the reaction kinetics with regard to a faster conversion (curing speed), (ii) the selected vulcanization temperature initiates the rapid start of the crosslinking reaction and affects the resulting sulfur network, and (iii) the quality of a part is ultimately affected by a different sulfur network.

The Rubber Process Analyzer (RPA) is mainly employed in industry to record the onset and progress of the crosslinking reaction. It is a dynamic–mechanical testing device consists of a biconical test chamber to achieve an almost homogeneous distribution of shear deformation. To characterize the isothermal crosslinking reaction, the test system is first heated, e.g., to 160 °C; then, the specimen is loaded, and the measurement starts. During the test, the lower test chamber is subjected to a sinusoidal strain oscillation. At the same time, the torque transmitted by the sample is detected and recorded by the upper test chamber [20,21]. Currently, the state-of-the-art practice is to normalize the curing characteristics, determined indirectly via the transmitted torque, at several isothermal temperatures. This involves normalizing each isotherm between 0 (non-crosslinked) and 1 (fully crosslinked), disregarding any differences in the minimum and maximum transmitted torque. On the one hand, this is carried out to define suitable manufacturing parameters, e.g., mold temperature and vulcanization time, and, on the other hand, to approximate the reaction kinetics with suitable models in order to enable the calculation of the degree of cure by means of a simulation routine.

Simulation routines have been successfully applied in the polymer sector for decades to virtually optimize real manufacturing processes [22]. These optimizations include: (i) the basic polymer-compatible part design, (ii) the choice of injection point, injection system, and number of cavities, (iii) the design of the complex injection mold, including the selection and type of the temperature control system, and (iv) the virtual prediction of the operation point. These virtual representations of real manufacturing processes can be considered, for example, to reduce the cost of any changes to the complex injection mold and to minimize the amount of material used when sampling the mold based on an already virtually optimized operating point. Although the utilization of simulation routines is already state of the art and has been in application for several decades, there are still challenges to be solved, especially in the area of reactive materials such as elastomers. The reaction kinetics is normally only estimated indirectly via the transmitted torque under isothermal conditions, which do not occur in real processes. In addition, the difference in the maximum transmitted torque that arises from different vulcanization temperatures is neglected by the normalization step. The associated loss of information is known, but it is completely ignored in the approximation. Furthermore, a filling and curing simulation is not carried out to directly compute the part quality, e.g., the permanent deformation, the dynamic–mechanical properties, or the fracture-mechanical behavior, but rather the degree of cure. The degree of cure is then either correlated with part quality depending on the operator or the quality is estimated by exporting relevant information with the help of other calculation programs. However, the processing history, i.e., the speed of the crosslinking reaction, which is largely responsible for the formation of poly-, di-, and monosulfidic network sites, for example, is not taken into account. Notwithstanding, this is of great importance in real production, as it significantly influences the final product properties.

This work continues the preliminary findings of Hornbachner [23] as well as of Traintinger et al. [12] and addresses precisely the challenges mentioned above by first manufacturing and characterizing parts with different degrees of cure. This was conducted to highlight the reliability of the assumptions made on the basis of normalized reaction isotherms with regard to part quality. Based on this, an innovative modeling approach was developed to provide, for the first time, the possibility of calculating part quality directly during filling and curing simulation, taking into account the processing history.

## 2. Theoretical Insights into an Innovative Modeling Approach

Especially in the area of process simulation of thick-walled parts, such as some elastomer products, a homogeneous temperature distribution cannot be assumed. Due to the low thermal conductivity of filled rubber compounds, i.e., $0.15\;W\;m^{-1}\;K^{-1}$–$0.40\;W\;m^{-1}\;K^{-1}$ in common manufacturing temperature ranges [24,25], huge temperature differences occur within the molded part during the crosslinking reaction. The material in contact with the hot mold surface therefore has a significantly higher temperature in comparison to the material in the center of the thick-walled part. These temperature inhomogeneities not only trigger the start of the crosslinking reaction at different times, but also significantly influence its speed. As a result, the crosslinking reaction does not take place at a constant speed throughout the whole part, but it takes place at different speeds at each volume element.

**Figure 1:**
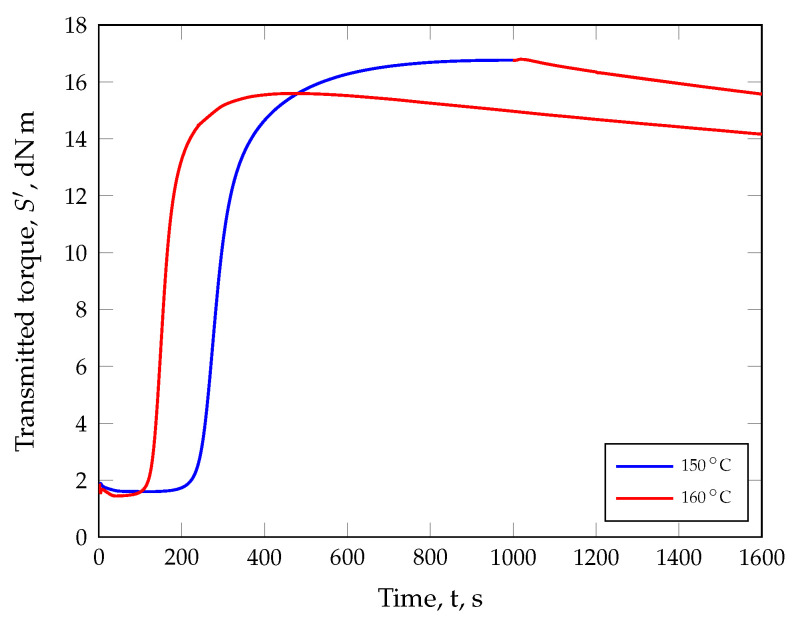
Difference in the isothermal crosslinking behavior of a sulfur-based styrene butadiene rubber at a temperature of 150 °C (blue curve) followed by a temperature increase of 10 K for 600 s as well as at a temperature of 160 °C (red curve).

While developing the present innovative modeling approach, the following question arose: If a crosslinking reaction is carried out under ideal conditions with a Rubber Process Analyzer (RPA) at an isothermal temperature of 150 °C until the plateau is reached and then the temperature rises by 10 K for 600 s, is it possible to achieve the same transmitted torque $S^{\prime }$ with an isothermal analysis of 160 °C at the end of the measurement? As can be seen in Figure 1, this is not the case. After applying the two temperature programs, there is a difference between 150 °C and 160 °C in the maximum transmitted torque of 1.16 dNm (7%) while, after the temperature rise, the difference consists of 1.39 dNm (9%) at the end of the analysis. Despite the fact that the same material was tested and the temperature was constant for a longer period of time at the end of the experiment, the mechanical response was different.

Moreover, this material behavior is still ignored in state-of-the-art models of reaction kinetics. Although it is known that the maximum transmitted torque decreases with increasing test temperature, as shown in Figure 1 and observed by Khang et al. [26] for natural rubber as well as by Hornbachner [23] for styrene–butadiene rubber and ethylene propylene diene rubber, for example, these observations are first normalized and then approximated according to models by Deng Isayev [27,28] or Kamal and Sourour [29]. The associated loss of mechanical information based on the normalization step is accepted.

As a result, it is not recommended to correlate a degree of cure simulated on the basis of these assumptions with a part quality, e.g., the compression set.

To overcome these challenges, Weinhold proposed a phenomenological modeling approach. This approach includes the average curing speed $\bar{\dot{c}}\left(t,T\right)$ to optimally take into account the process history, i.e., the locally different temperature development impacts the speed of the crosslinking reaction and thus the associated mechanical material response. The average curing speed (ACS) model is given by Equation (1) and the boundary condition by Equation (2).

(1) $$\begin{array}{c} \bar{\dot{c}}\left(t,T\right)=\frac{1}{t_{max}}\int_{t_{i}}^{t_{max}}\frac{\partial }{\partial t}c\left(t,T\right)\;dt \end{array}$$

(2) $$\begin{array}{c} c_{min}\left(t_{i},T\right)\;\leq \;c\left(t,T\right)\;\leq \;c_{max}\left(t_{max},T\right) \end{array}$$

The restriction to a minimum $c_{min}\left(t_{i},T\right)$ and maximum $c_{max}\left(t_{max},T\right)$ degree of cure c(t,T) ensures that only the ongoing curing reaction, i.e., from the incubation time $t_{i}$ at a given temperature *T* to the maximum degree of cure $c_{max}\left(t_{max},T\right)$ at time $t_{max}$, is taken into account. All times before $t_{i}$ and after the main curing reaction, i.e., when a degree of cure of 100% is reached (for example, reversion or marching modulus), are neglected in the calculation. The average curing speed therefore captures the temperature-dependent curing speed during the vulcanization process. At isothermal temperatures, i.e., given during the characterization of the curing reaction with an RPA, the average curing speed is simplified to the secant gradient from the start of curing to its end.

**Figure 2:**
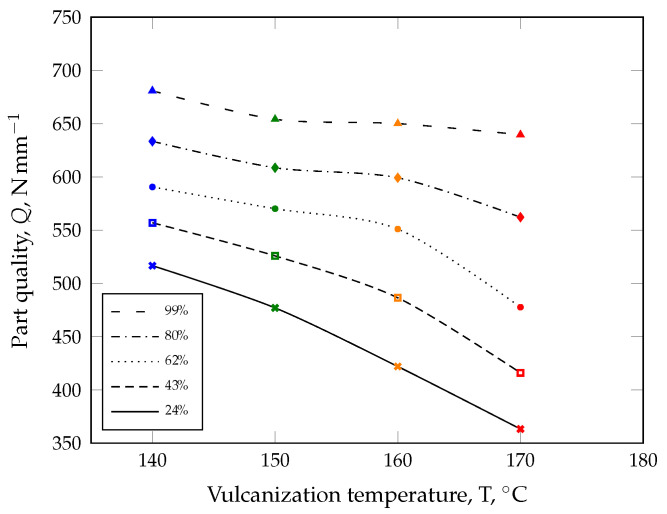
The vulcanization temperature has a considerable influence on the part quality (symbols), even if they were manufactured to the same degree of cure (trend lines).

However, constant mass temperature cannot be assumed in real production processes. Consequently, the ACS model must be calibrated once with part quality parameters. Initially, the calibration process requires that parts are manufactured at different mold temperatures and curing times in order to ensure various curing states. After this step, all manufactured parts are subjected to quality control (Figure 2). The part quality can either be the compression set, for example, the tensile test, derived from dynamic–mechanical analysis, such as the dynamic spring constant [30], or any other quality-relevant parameter.

Finally, Equation (1) can be applied to calculate the average curing speed based on isothermal curing characteristics. In simple terms, the secant slope is calculated based on RPA measurements at each manufacturing temperature between $t_{i}$ and the production time corresponding to the degree of cure. This procedure will be carried out for all settings.

**Figure 3:**
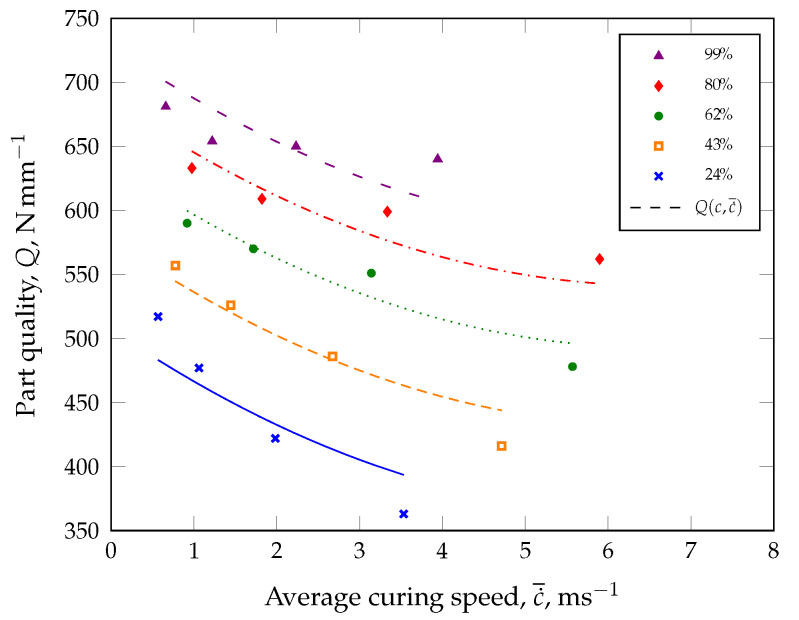
Comparison of determined part quality *Q* and quadratic approximation $Q(c,\bar{\dot{c}})$ (adjusted $R^{2}$ of 87%) as a function of the degree of cure *c* and the average curing speed $\bar{\dot{c}}$. In the demonstration case, the dynamic spring constant from a dynamic–mechanical analysis in compression mode was considered as a quality-relevant parameter.

Instead of including the temperature as a parameter in the innovative ACS model, the part quality will be plotted as a function of the average curing speed and subsequently approximated with a suitable model (see Figure 3).

For this purpose, an approximation model, e.g., a model with the lowest standard error of the regression, should be selected for the numerical approximation of the part quality. As an example, a quadratic model (Equation (3)) is proposed for calculating the part quality in the demonstration case (Figure 3) employing a quality parameter *Q*.

(3) $$\begin{array}{c} Q\left(c,\bar{\dot{c}}\right)=\alpha +\beta_{1}c\;+\;\beta_{2}c^{2}\;+\;\gamma_{1}\bar{\dot{c}}\;+\;\gamma_{2}{\bar{\dot{c}}}^{2} \end{array}$$

Here, the model parameters α, $\beta_{i}$, and $\gamma_{i}$ with the indices i=1,2 are estimated with the method of minimizing the error squares. With the model parameters specified in Table 1, the quadratic model is able to describe the characterized part quality behavior very well with an adjusted coefficient of determination of $R^{2}=$ 87%, as shown in Figure 3 (dashed curve).

All of the aforementioned steps are essential for the accurate simulation of part quality based on the processing history. The locally resolved degree of cure and the average curing speed can be calculated with the aid of a process simulation, i.e., the ACS model is fully implemented in the SIGMASOFT^®^ v6.0 (SIGMA Engineering GmbH, Aachen, Germany) simulation routine, and subsequently serve as input variables in the innovative ACS model. As a result, the locally resolved part quality is plotted instead of the degree of cure, which replaces the homogeneous mechanical quality criteria for structural analysis.

## 3. Materials and Methods

This section provides an overview of (i) the rubber compound subjected to investigation, (ii) the analysis of reaction kinetics, (iii) the manufacturing process for part production, (iv) the compression set testing procedure, and (v) the setup of a rubber compression molding simulation incorporating the innovative modeling approach of the average curing speed (ACS) model. This model is utilized for predictive virtual assessment of part quality, taking into account the material’s processing history.

### 3.1. Materials

An industrial-scale styrene butadiene rubber (SBR) compound was provided by Semperit Technische Produkte Gesellschaft m.b.H (Wimpassing, Austria) and used for all tests conducted in the course of this work. This material contains a sulfur-based crosslinking system, it is reinforced with carbon black and white fillers, and it has a Shore A hardness of 71 after being cured. Notably, the material’s application suitability is highlighted by its exceptional damping properties between −20 °C and 60 °C, as documented in previous research [31]. For reasons of confidentiality, the exact composition of the compound cannot be revealed in detail.

### 3.2. Reaction Kinetics

A Rubber Process Analyzer (RPA) D-MDR 3000 (MonTech Werkstoffpruefmaschinen GmbH, Buchen, Germany) was utilized to determine the crosslinking isotherms or the time evolution of the crosslinking reaction at temperatures between 140 °C and 170 °C in 10 K steps. To validate the ACS model, reaction kinetics at 155 °C and 165 °C were additionally analysed with the RPA following DIN 53529 [20,21] standard. During the characterization, the torque transmitted in phase ($S^{\prime }$) is recorded (test frequency 1.67 Hz, amplitude 0.5°, and test time *t* to reach at least the maximum of $S^{\prime }$, e.g., 1 h) and then normalized. At this point, it should be mentioned that the investigated SBR exhibits no marching modulus, e.g., a continuous increase in $S^{\prime }$. For the normalization step of each isothermal curing behavior (Equation (4), the maximum of $S^{\prime }$ is considered as fully cured ($c=100\%\;\;@\;S_{max}^{\prime }$) and the minimum of $S^{\prime }$ after the running in phase as uncured ($c=0\%\;\;@\;S_{min}^{\prime }$).

(4) $$\begin{array}{c} c=100\frac{S^{\prime }-S_{min}^{\prime }}{S_{max}^{\prime }-S_{min}^{\prime }} \end{array}$$

Based on these normalized reaction isotherms, five equidistant conditions between low and high (99%) degrees of cure were selected for each isotherm to subsequently manufacture compression molded parts. The minimum possible degree of cure or the corresponding heating time could only be finally defined during part manufacturing. This is due to the low thermal conductivity of rubber materials [24,25] and the associated significantly lower degree of cure of thick-walled parts in relation to the reaction kinetics obtained with the help of a Rubber Process Analyzer (RPA, sample thickness in the inner area of 0.5 mm). In production, insufficient crosslinking leads to the challenge that molded parts may no longer be demolded.

### 3.3. Manufacturing of Compression Molded Parts

**Figure 4:**
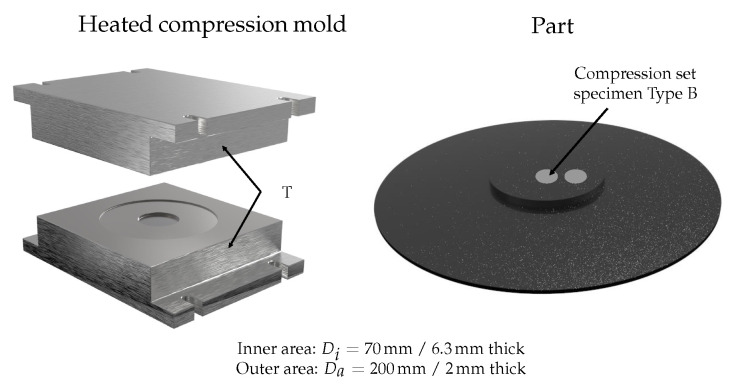
Applied mold for manufacturing of compression molded parts, part geometry, and test positions.

**Figure 5:**
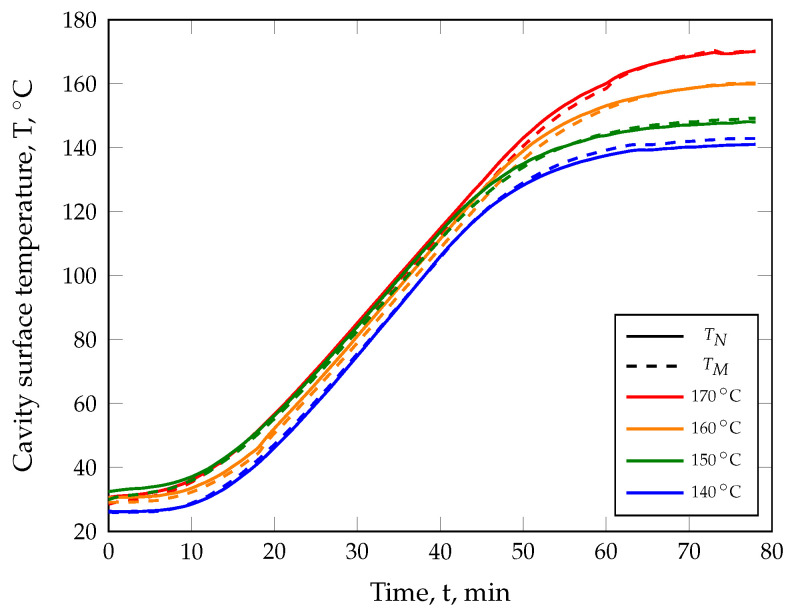
Nozzle ($T_{N}$) and moving ($T_{M}$) cavity surface temperature during the heating process of the compression mold to ensure the required mold temperature.

The manufacturing of compression molded parts was carried out with a rubber injection molding machine MTF750/160editionS (MAPLAN GmbH, Kottingbrunn, Austria) with the aid of a compression mold that can be indirectly heated via the machine clamping plates (Figure 4). Since thermocouples for temperature monitoring and control are not available in this mold, the temperature was recorded with temporarily installed and fast-response type K thermocouple wires to ensure that the required cavity surface temperature, e.g., 140 °C, was achieved. The cavity surface temperatures recorded on the nozzle and moving platen sides of the cavity during the heating process of the compression mold are shown in Figure 5, whereas the machine clamping platen temperatures adjusted on the injection molding machine to reach the required cavity surface temperature are listed in Table 2. As a result, the defined surface temperature was reached with a deviation of ±1K after the mold preheating process.

Subsequently, compression molded parts were manufactured with a preform (68 mm side length, 20 mm in thickness) at a vulcanization temperature of 140 °C and at various heating times based on the reaction isotherms. The process handling times listed in Table 3 were maintained throughout the entire experiments. This enabled the lowest heating time to be determined, at which point it was still possible to demold the part. For all other manufacturing temperatures, the minimum heating time determined at 140 °C or the corresponding degree of cure (24%) read off from the reaction kinetics at 140 °C was thus defined as the minimum level for all other temperatures as well. In total, three parts were produced for each vulcanization time (Table 4), whereby one part was manufactured with a long heating time, followed by one with a short heating time, in order to prevent the mold from cooling down due to the indirect temperature control. For validation purposes of the ACS model, additional parts were fabricated with a degree of cure of 80% at 155 °C and at 165 °C, which was read off from the reaction kinetics. The corresponding vulcanization times were 341 s and 189 s, and the temperatures of the nozzle side as well as of the moving platen side of the clamping plates were set to 161 °C and 172 °C. To capture the degree of cure after manufacturing, all parts were immediately placed in ice water and cooled.

### 3.4. Characterization of Part Quality

In the rubber industry, it is common to examine the degree of cure of a manufactured part by means of compression set (CS), as this method is the most accepted. For this purpose, two CS specimens were extracted from each manufactured part (in the center and next to it, cf. Figure 4), followed by CS testing according to DIN ISO 815 [32] standard, i.e., test specimen Type B with a dimension of ⌀13 mm±0.5 mm and 6.3 mm±0.3 mm, compression of 25±2%, and stored at 70 °C±1 °C for 24 h. 24−2+0h h.

After this aging step, the compression was released, the samples were stored on a plate for 30 min±3 min under standard laboratory conditions to cool down, and subsequently the height of the sample was measured again. With the aid of Equation (5), CS is calculated [32]:

(5) $$\begin{array}{c} CS=100\frac{h_{0}-h_{r}}{h_{0}-h_{c}}, \end{array}$$

where $h_{0}$, $h_{c}$, and $h_{r}$ represent the initial and compressed height as well as the height after the relaxation phase. During CS testing of real molded parts, values for CS exceeding 100% may be calculated. Such cases have been observed mainly on parts with a very low degree of cure in the core area. While it was possible to demold them, since the vulcanization of the surface layers was already advanced, the material behavior in the core was still predominantly viscous. Accordingly, the specimen does not relax after compression unloading. Instead, the material flows or shrinks due to cooling. As a result, smaller values for $h_{r}$ are obtained. However, since $h_{r}$ must be greater than $h_{c}$, these values are not reasonable and should not be considered in further interpretation.

### 3.5. Simulation

**Figure 6:**
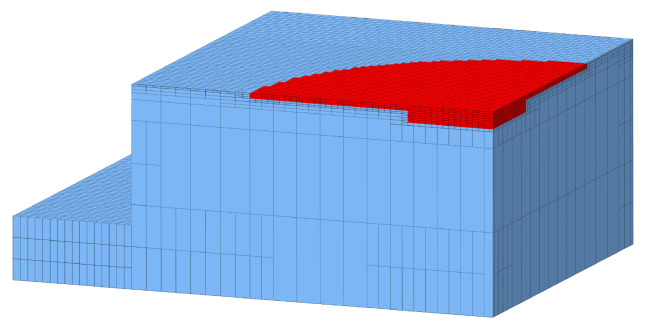
Section view of the 3D tetra mesh of the moving mold half and the rubber part. Element coarsening was set for the mold, whereas a finer mesh containing 10 equidistant elements in thickness direction were selected for the mid area of the rubber part.

The real compression molding process was modeled by means of the simulation routine SIGMASOFT^®^ v6.0 (SIGMA Engineering GmbH, Aachen, Germany). In this context, the computer-aided design (CAD) of the compression mold and part (cf. Figure 4) were imported and the mesh was defined individually for both. While 10 equidistant elements along the thickness direction were selected for the mid area of the part, a coarsening definition was selected for the mold. Figure 6 sketches a sectional view of the meshed setup.

Although not explicitly shown, but important for the interpretation of the simulation results, a so-called evaluation area was defined in the center of the part. This area marks a domain, which is exclusively intended for the calculation of user-defined results, but neither influences the mesh generation nor the simulation. In general, the dimensions of the evaluation area can be set arbitrarily, while in this work the shape of a CS sample was selected. Similar to the real compression molding process, a preform with a dimension of 68 mm×68 mm×20 mm and an initial mass temperature of 25 °C was specified for the virtual design and positioned centrally in the moving mold half. In the next step, (i) a steel of grade 1.2312 was assigned to the mold, (ii) the material data shown in Appendix A were linked to the rubber part, and (iii) heat transfer coefficients (HTCs) for the contact between steel and steel ($10\;kW\;m^{-2}\;K^{-1}$) as well as between rubber and steel ($0.8\;kW\;m^{-2}\;K^{-1}$) were defined. Finally, (iv) the moving mold closing speed profile obtained from the real process (Table 5), and (v) selected vulcanization times in combination with process handling times from Table 3 were considered.

It is important to mention that the ACS model needs to be set active before starting the simulation. This enables the calculation of user-defined results based on Equation (3) and the procedure described in Section 2.

## 4. Results and Discussion

This section provides insights into (i) the reaction kinetics of the SBR, (ii) the part properties, and (iii) the application and validation of the average curing speed model to predict the part performance taking into account the processing history. All measured data concerning reaction kinetics (state of cure for each temperature) and part properties (compression set for each manufacturing temperature under each state of cure) are available in the Supplementary File.

### 4.1. Reaction Kinetics of the SBR

**Figure 7:**
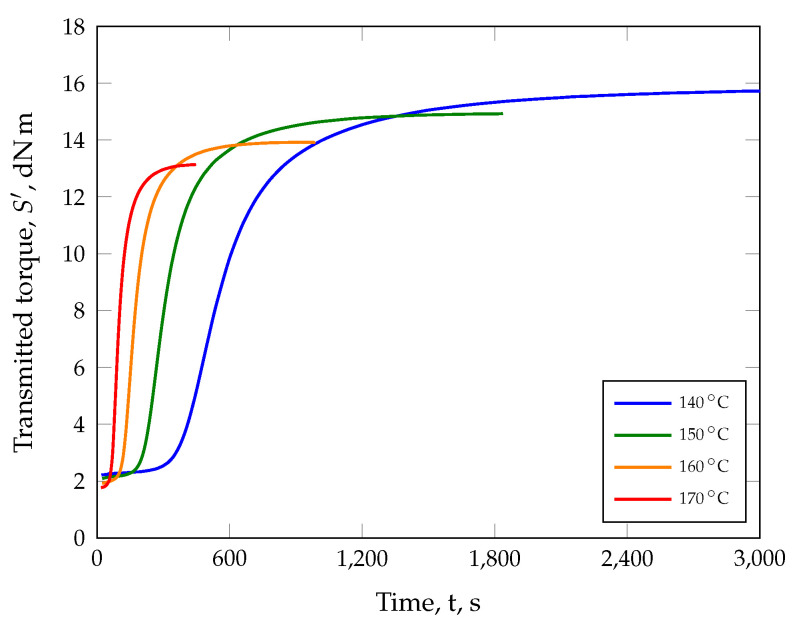
A temperature increase of 30 K leads to a reduction in the incubation time by 230 s with a simultaneous decrease in the maximum transmitted torque by 2.65 dN m (17%) as well as in the minimum transmitted torque by 0.45 dN m (20%).

Prior to the manufacturing of compression-molded parts, the isothermal crosslinking behavior was characterized for several temperatures with the help of an RPA (Figure 7).

**Figure 8:**
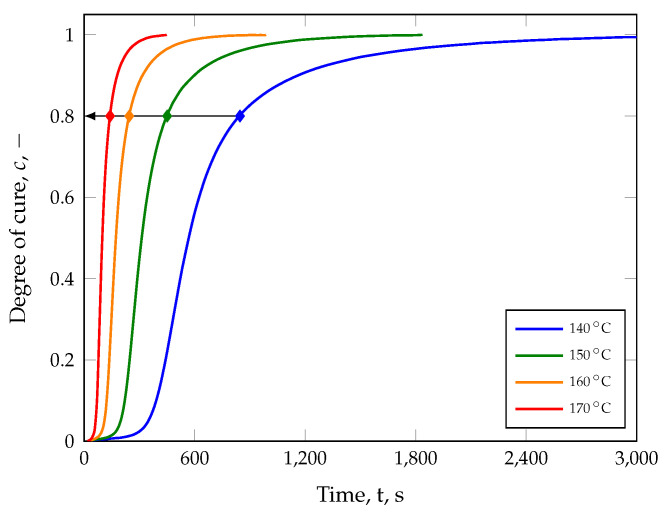
Normalized isotherms of the transmitted torque to illustrate the crosslinking characteristics and selection of suitable manufacturing times (see Table 4). According to theory, the same degree of cure will be achieved by increasing the temperature and reducing the vulcanization time (diamond symbol).

It can be seen that an increase of 30 K leads to a reduction in the incubation time by 230 s with a simultaneous decrease in the maximum transmitted torque by 2.65 dN m (17%) as well as in the minimum transmitted torque by 0.45 dN m (20%). The state-of-the-art technique is now to normalize these data by applying Equation (4) and plotting it (Figure 8). On the one hand, this enables the visualization of the crosslinking behavior at different temperatures and the selection of suitable vulcanization times (cf. Table 4) and, on the other hand, the approximation of the reaction kinetics via suitable models, to include the temperature-dependent curing behavior in the simulation. Consequently, parts produced at 140 °C for 846 s exhibit the same degree of cure (80%) as those parts manufactured at 170 °C for 141 s. However, in theory, only the selected vulcanization time is relevant in order to achieve the same degree of cure when the vulcanization temperature is increased. Whether the loss of information regarding the normalization procedure, i.e., different minima and maxima in the transmitted torque will be neglected, has an effect on the properties of compression molded parts will be shown later.

### 4.2. Part Properties

**Figure 9:**
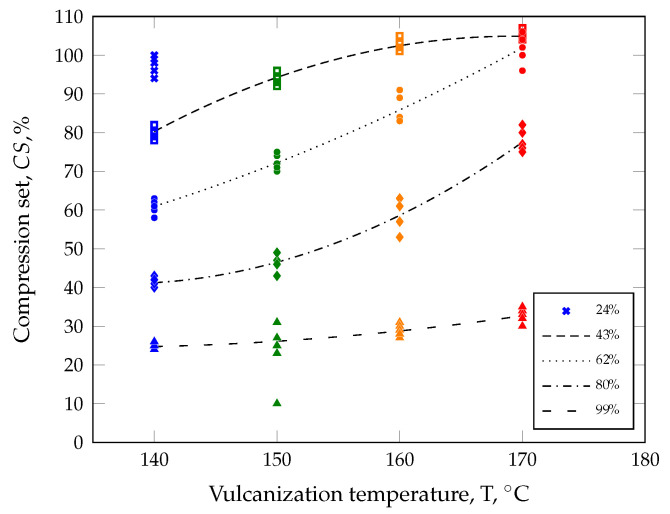
Quality test results (symbols) of parts compression molded at different vulcanization temperatures. It can be seen that the parts show considerable differences in compression set at different vulcanization temperatures, although they should exhibit the same degree of cure (trend lines) based on the normalized transmitted torque isotherms.

The results of the quality test of all compression-molded parts are given in Figure 9. For parts with a degree of cure of 24%, meaningful compression set values could only be determined for a vulcanization temperature of 140 °C. At all higher temperatures, the parts could be demolded. However, the quality tests resulted in CS values of ≫ 100%. As described in the Methods Section, these values are irrelevant and have been excluded from further interpretation.

It is well known that prolonging curing at a constant temperature reduces CS, e.g., at 140 °C from CS = 97% (*c* = 24%) to CS = 25% at *c* = 99%.

In sulfur-based rubber systems, it is well known that the polysulfidic crosslinks dissociate during prolonged vulcanization and induce the formation of di- and monosulfidic bonds between the polymer chains [12,33,34]. Consequently, the material behaves more elastically and the permanent deformation, also known as compression set, is lower after a compression load. However, if the vulcanization temperature at a defined degree of cure of 99% is increased from 140 °C to 170 °C, the CS values are not constant, as assumed before, but increase from 25 ±1% to 33 ±2%. This corresponds to a relative increase in the CS of 32%, i.e., a reduced ability of the material to recover after a compression load. A more pronounced behavior is observed at a selected degree of cure of 80%, resulting in CS values of 41−1+2% at 140 °C and 77−2+5% at 170 °C. This difference corresponds to a factor of approx. 1.8. In the case of 62% and 43%, a similar trend can be observed. A crucial point, however, relates to the fact that it is not possible to assign a degree of cure exclusively to one part quality. CS measurements of parts produced at 170 °C (c=80%) result in CS =77−2+5%. A very similar quality value of CS =77−1+4% can be achieved with the setting 150 °C (c=62%).

As already discussed, loss of information occurs when evaluating the CS measurement results if the vulcanization times are selected exclusively on the basis of normalized transmitted torque isotherms. In other words, if the vulcanization time is reduced and at the same time the vulcanization temperature is raised in order to achieve the same degree of cure (cf. Figure 8). Whether this loss of information can be explained solely by the normalization of the isotherms of the transmitted torque remains to be seen. Another important factor is the difference in thickness between the RPA specimen and the real part. Due to thicker parts compared to RPA specimens and the low thermal conductivity of the SBR ($0.284\;W\;m^{-1}K^{-1}$), isothermal conditions in each volume element cannot be assumed. Instead, the surface layer of the part is essentially at the set mold temperature, for example, at 160 °C or 170 °C, throughout the entire crosslinking reaction, while the cold core remains more or less at room temperature at the start of vulcanization, i.e., especially in the case of compression molding, where negligible shear heating occurs during cavity filling. As a result of these extreme temperature differences, the curing speed in each volume element varies considerably, resulting in a distribution of the degree of cure in the part. Traintinger et al. [12] have demonstrated these effects simulatively for two settings of this test series. For both, the targeted degree of cure read off from the normalized transmitted torque isotherms was defined as 80%. In the evaluation area, e.g., volume of the CS sample, setting one (160 °C and 245 s) resulted in an average simulated degree of cure of 42%, whereas setting two (170 °C and 141 s) led to an average of only 10%. This significant difference in the degree of cure helps to explain the fact that the quality test of the compression-molded parts cannot yield constant values for CS. Furthermore, a variation in temperature in each volume element triggers the curing speed in a different way. As a result, polysulfidic and ultimately di- and monosulfidic network sites form in a first step directly proportional to the curing speed. Thus, a different vulcanization temperature, e.g., 160 °C vs. 170 °C, leads to a change in the network structure at the end of the reaction and in turn impacts all quality criteria decisive for rubber products, such as compression set and dynamic mechanical behavior, for example [12].

In summary, temperature is not a suitable parameter for describing part quality. Thus, the following section provides a detailed explanation of the average curing speed model to address these challenges.

## 5. Application and Validation of the Average Curing Speed Model

As described in Section 2, the coefficients required for the ACS model were first calculated; then, the part quality was simulated for two selected settings, and finally compared with the results obtained for compression-molded parts.

**Figure 10:**
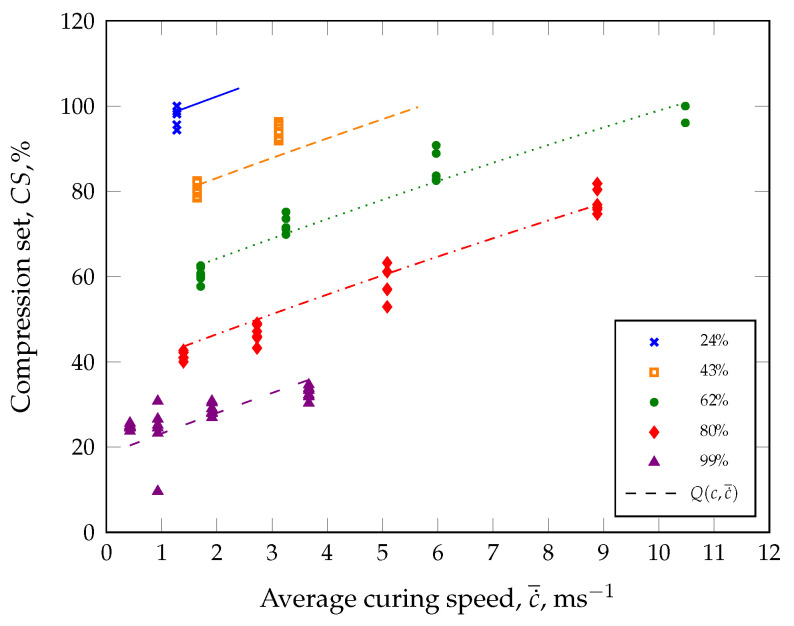
Comparison of compression set results and quadratic approximation $CS(c,\bar{\dot{c}})$ (adjusted $R^{2}$ of 95%) as a function of the degree of cure *c* and the average curing speed $\bar{\dot{c}}$.

For the initial calibration of the ACS model, the average curing speed $\bar{\dot{c}}$ was calculated as the secant slope based on the reaction kinetics shown in Figure 8 and for each vulcanization time (see Table 4). For example, the secant slope was computed as $1.396\;ms^{-1}$ for a part manufactured at 140 °C with a defined degree of cure of 80%. In this case, it should be noted that the incubation time was selected at a degree of cure of 2%. However, approximation of the compression set values was achieved from the calculated average curing speeds $\bar{\dot{c}}$, degree of cure *c*, and Equation (3). Figure 10 shows that $CS(c,\bar{\dot{c}})$ describes the measured data with an adjusted $R^{2}$ of 95%.

Subsequently, a filling and curing simulation was carried out applying the ACS model parameters shown in Table 6.

**Figure 11:**
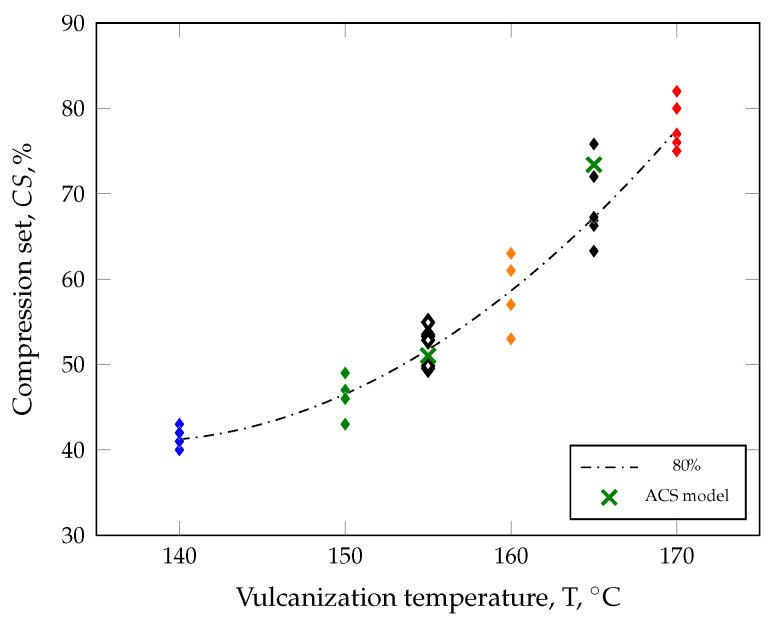
The innovative average curing speed (ACS) model was applied in the simulation, and the results for a degree of cure of 80% showed excellent prediction of part quality (represented by black diamond symbols) compared to the ACS model’s output (mean values represented by green crosses). Colored diamond symbols indicate CS values for other vulcanzation temperatures.

To verify the applicability of the ACS model, Figure 11 compares the CS values of all parts manufactured at different vulcanization temperatures and a defined degree of cure of 80%.

The permanent deformation median values for parts vulcanized at 155 °C and 165 °C are 53−4+2% and 67−4+9% (black diamonds), respectively, and are in line with the expected trend.

**Figure 12:**
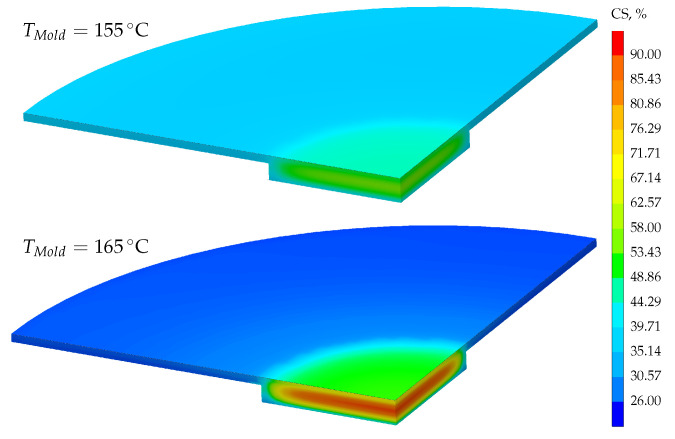
The simulated part quality is based on the innovative average curing speed (ACS) model, which considers the processing history. For the vulcanization temperature and time, 155 °C for 341 s (**top**) and 165 °C for 189 s (**bottom**) were selected.

Overall, the average part quality simulated with the innovative average curing speed model provides an excellent prediction of the real molding process. The predicted mean CS values (green crosses) of the evaluation area, i.e., volume of a CS specimen, are 51% and 73% as well as the absolute deviation from the median of the characterized parts is $\Delta CS_{155\;{}^{\circ}C}=2$% and $\Delta CS_{165\;{}^{\circ}C}=6$%. In addition, the simulated CS values are within the range of the tested parts, confirming the applicability of the ACS model. Another advantage of the ACS model is shown in Figure 12. To assess the part quality, it is no longer necessary to assume the part quality based on the simulated degree of cure. Instead, the quality can be mapped individually for each volume element, which will enable new optimization strategies for part design in the future.

To conclude, the validation highlighted the enormous potential of the innovative modeling approach. Beyond this, it was shown that the curing speed is a suitable measure for the quality simulation of the compression molding process. However, to discuss possible limitations of the model, in particular the approximation of part quality as a function of the degree of cure and average curing speed based on a quadratic model, the authors investigated the more complex rubber injection molding process [35]. It turned out that, in this case, a quadratic model was not suitable for the approximation. Instead, a logistic model was chosen to link the part quality to the degree of cure and the average curing speed. It should be noted that any approximation model can be chosen, but only the one with the lowest standard error of regression should be considered. Another limitation could be that there is no suitable model to approximate the part quality for a really unusual material behavior, e.g., by considering material blends or when there is no change in part quality depending on the degree of cure. Furthermore, the authors limited their investigations to sulfur-based crosslinking systems. In further studies, peroxide- or bisphenol A-based crosslinking systems have to be considered in order to ensure the general validity of the innovative modeling approach. Overall, it was demonstrated that the model is valid for both the compression molding process as well as for the complex injection molding process, ensuring the transferability of application and enabling the processing history to be taken into account in the calculation of part quality for the first time.

## 6. Conclusions

This work presented a novel modeling approach for the direct prediction of mechanical properties in compression-molded rubber parts, shifting the focus from traditional degree-of-cure-based methods to a quality-centric perspective. The proposed average curing speed (ACS) model incorporates both the degree of cure and curing speed, addressing limitations of existing reaction kinetics models that do not account for the influence of vulcanization temperature and time on the quality of the part. Validation of the ACS model demonstrated its ability to accurately predict compression set values across different processing conditions, with predicted results aligning closely with experimental measurements.

By integrating the ACS model into the SIGMASOFT^®^ simulation software (v6.0), this approach enables the mapping of quality predictions at the element level within the molded parts, overcoming the manual or external post-processing steps required by traditional methods. This advancement offers a significant step forward in optimizing part design and manufacturing processes by directly linking simulation outputs to end-use performance. The results show the potential of the ACS model as a reliable tool to improve the accuracy of simulation in rubber manufacturing. Future studies will extend this approach to more complex processes to further broaden its industrial applicability.

## Figures and Tables

**Table 1:** Model parameters to link the part properties (quality parameter *Q* in the demonstration case) with the degree of cure and the average curing speed.

Model Parameter	Value	Unit
α	4.057 × 10^2^	$N\;{mm}^{-1}$
$\beta_{1}$	4.535 × 10^2^	$N\;{mm}^{-1}$
$\beta_{2}$	−1.291 × 10^2^	$N\;{mm}^{-1}$
$\gamma_{1}$	−4.396 × 10^4^	$N\;{mm}^{-1}s$
$\gamma_{2}$	3.343 × 10^6^	$N\;{mm}^{-1}s^{2}$

**Table 2:** Set manufacturing temperatures of the mold clamping plates to reach the required mold temperatures.

Required Mold Temperature, °C	140	150	160	170
Nozzle side, °C	145	155	167	178
Moving platen side, °C	146	156	166	178

**Table 3:** Process handling times.

Process Phase	Time, s
Loading the compound	2.0
Close mold	16.0
Open mold	5.7
Eject molded part	3.0
Total handling time	26.7

**Table 4:** Vulcanization times in s for the manufacturing of compression molded parts.

Degree of Cure, %	Temperature, °C			
	140	150	160	170
24	460	256	142	81
43	535	296	163	94
62	635	348	191	110
80	846	451	245	141
99	2551	1207	598	317

**Table 5:** Mold closing and opening profile.

Position, mm	390	389	30	29.9	0
Velocity, $mm\;s^{-1}$	0.1	75	75	5	5

**Table 6:** Model parameters to link the compression set values with the degree of cure and the average curing speed.

Model Parameter	Value	Unit
α	1.170 × 10^2^	%
$\beta_{1}$	−1.028 × 10^2^	%
$\beta_{2}$	3.063	%
$\gamma_{1}$	4.963 × 10^3^	%s
$\gamma_{2}$	−5.149 × 10^4^	%s^2^

**Figure A1:**
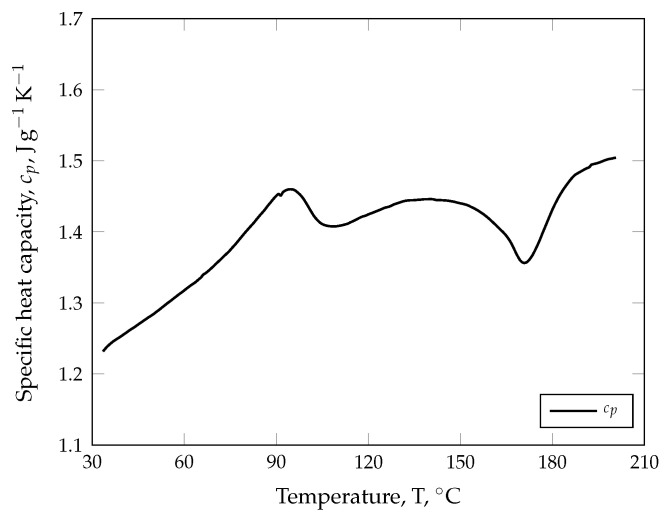
Calculated specific heat capacity ($c_{p}$) of SBR was obtained on a DSC employing a sapphire reference. The measurement was conducted with a heating rate of $10\;K\min^{-1}$ under a nitrogen atmosphere with a purge rate of $50\;mL\;\min^{-1}$.

## Data Availability

All data presented in this publication are only available upon request to the corresponding author, assuming a formal approval of the involved companies and co-authors.

## Supplementary Materials

The following supporting information can be downloaded at: https://www.mdpi.com/article/10.3390/polym17020149/s1.

## Appendix A

All material characterization of the SBR was undertaken internally in order to be able to define the material dataset in the simulation routine. Additionally, all measured material data such as reaction kinetics, viscosity, pressure-specific volume-temperature behavior, specific heat capacity, and thermal conductivity were transferred to the simulation routine SIGMASOFT^®^ v6.0 (SIGMA Engineering GmbH, Aachen, Germany) and subsequently approximated with suitable models.

Therefore, a Rubber Process Analyzer (described in Section 3.2) was applied to determine the reaction kinetics at 140 °C, 150 °C, 160 °C, and 170 °C. The transmitted isothermal torque data were normalized according to Equation (4) and approximated by the approach of Kamal and Sourour [29]. This leads to the model parameters for the incubation time: (i) $log_{10}\left(t_{0}\right)=-10.052\;\min$ and (ii) $T_{0}=10188.615\;K$. In addition, the model parameters for the reaction kinetics are (i) $log_{10}\left(A_{1}\right)=-5.999\;s^{-1}$, (ii) $log_{10}\left(A_{2}\right)=10.073\;s^{-1}$, (iii) $E_{1}=1.680\;\times \;10^{-2}\;J\;{\mathrm{mol}}^{-1}$, (iii) $E_{2}=94.760\;kJ\;{\mathrm{mol}}^{-1}$, (iv) n=0.909, and m=1.712.

On the same device, the flow behavior in the low shear range, i.e., <$300\;\mathrm{rad}s^{-1}$, was characterized at 60 °C, 80 °C, and 100 °C with the following settings: (i) pre-heating of the sample for 4 min (amplitude of 3°, frequency of 5 Hz) followed by (ii) a frequency sweep in the range of 45 Hz to 0.5 Hz with a constant amplitude of 0.1°. Afterwards, the obtained viscosity data were approximated with the Cross–Williams–Landel–Ferry (Cross-WLF) model [36,37] leading to the following approximation parameters: (i) $A_{1}=16.653$, (ii) $A_{2}=167.666\;K$, (iii) n=0.194, (iv) $D_{1}=3.587\;\mathrm{MPa}s$, (v) $D_{2}=353.150\;K$, (vi) $D_{3}=0.000\;K{\mathrm{Pa}}^{-1}$, and (vii) $D_{4}=0.204\;\mathrm{MPa}$.

The specific volume was analyzed in dependence on pressure and temperature (pvT) in isobaric heating mode ($0.1\;Ks^{-1}$) between 40 °C and 200 °C at pressure levels ranging from 20 MPa to 160 MPa in 20 MPa increments. For this test, a PVT-100 device (SWO Polymertechnik GmbH, Krefeld, Germany) was utilized and the obtained pvT-data were approximated with the two-domain model of Tait [38,39]. All model parameters are given in Table A1.

**Table A1:** Model parameters for SBR in the low-temperature region (s), high-temperature region (m), and transition region of the two-domain pressure-specific volume-temperature (pvT) model of Tait.

Low			High		
$b_{1s}$	$7.738\;\times \;10^{-4}$	$m^{3}{kg}^{-1}$	$b_{1m}$	$7.728\;\times \;10^{-3}$	$m^{3}{kg}^{-1}$
$b_{2s}$	$2.612\;\times \;10^{-7}$	$m^{3}{kg}^{-1}\;K^{-1}$	$b_{2m}$	$3.449\;\times \;10^{-7}$	$m^{3}{kg}^{-1}\;K^{-1}$
$b_{3s}$	$2.052\;\times \;10^{8}$	Pa	$b_{3m}$	$2.055\;\times \;10^{8}$	Pa
$b_{4s}$	$1.452\;\times \;10^{-3}$	$K^{-1}$	$b_{4m}$	$4.054\;\times \;10^{-3}$	$K^{-1}$
$b_{7}$	0	$m^{3}{kg}^{-1}$	**Limit**		
$b_{8}$	0	${kg}^{-1}$	$b_{5}$	$1.063\;\times \;10^{-2}$	°C
$b_{9}$	0	${\mathrm{Pa}}^{-1}$	$b_{6}$	$1.440\;\times \;10^{-7}$	$K\;{\mathrm{Pa}}^{-1}$

The specific heat capacity was determined by means of a differential scanning calorimeter DSC1 STAR^e^ System (Mettler-Toledo GmbH, Greifensee, Switzerland). To ensure excellent contact between the sample and the crucible, the rubber was compression-molded into a 1 mm thick sheet at room temperature and a sample of ⌀5 mm was punched out. The specific heat capacity shown in Figure A1 was recorded at a heating rate of $10\;K\min^{-1}$ under a nitrogen atmosphere with a purge rate of $50\;mL\;\min^{-1}$ to inhibit oxidation reactions.

The thermal conductivity of a compression-molded sheet was tested with a guarded heat flow meter DTC300 (TA Instruments, New Castle, DE, USA). At 64 °C, the determined thermal conductivity is $0.284\;W\;m^{-1}\;K^{-1}$. It should be noted that the determination of the thermal conductivity λ at one temperature is sufficient, since λ is not a function of temperature in the processing-relevant range, as many scientists proved [24,25].
